# Laparoendoscopic Rendezvous for Concomitant Cholecystocholedocholithiasis: A Successful Modality Even in the Most Difficult Presentations Including Pregnancy

**DOI:** 10.1155/2016/8618512

**Published:** 2016-12-25

**Authors:** Bader Hamza Shirah, Zaher Abdulaziz Mikwar, Akram Neyaz Ahmad, Yaser Mohammed Dahlan

**Affiliations:** ^1^King Abdullah International Medical Research Center, King Saud bin Abdulaziz University for Health Sciences, Jeddah, Saudi Arabia; ^2^Department of Surgery, King Abdulaziz Medical City, Jeddah, Saudi Arabia; ^3^Department of Medicine, King Abdulaziz Medical City, Jeddah, Saudi Arabia

## Abstract

*Background*. Laparoendoscopic rendezvous (LERV) technique is emerging as an attractive treatment option for concomitant cholecystocholedocholithiasis. In this paper, we report our experience in performing the LERV technique in patients with unusual presentations in terms of anatomical difficulty, pregnancy, multiple comorbid diseases, and postlaparotomy. We aim to highlight the effectiveness of the LERV technique in some clinical situations where conventional methods would fail or carry high risks in adequately managing concomitant cholecystocholedocholithiasis.* Methods*. Four patients diagnosed to have concomitant cholecystocholedocholithiasis with associated difficult presentation or comorbid diseases were treated using the LERV technique. One patient presented with difficult anatomy where ERCP failed at initial attempts. Another patient was pregnant (first trimester). A third patient had complex comorbid diseases (bronchial asthma, hypertension, congestive heart failure, and end-stage renal disease on regular hemodialysis). A fourth patient had previous laparotomy and sigmoidectomy for diverticular disease and had severe hospital phobia.* Results*. All patients tolerated the LERV technique very well; no intraoperative occurrence was reported. The mean operative time was 86.3 ± 17.2 minutes; mean time of the endoscopic part was 29.4 ± 3.57 minutes. The mean blood loss was 44.3 ± 18.2 mL (range 20–85). Residual stone, postoperative complications, postoperative morbidity, and postoperative mortality were 0 (0%). Postoperative short hospital stay was reported in all patients, average 3 days (range 2–4).* Conclusion*. LERV procedure is a safe and effective treatment option for the management of concomitant cholecystocholedocholithiasis, even in difficult situations where other methods would fail or carry high risks, or in patients presenting with severe comorbid diseases or pregnancy. This procedure may emerge as an attractive alternative option for high-risk patients. A patient's wishes may also influence the selection of this procedure. More scientific studies recruiting more patients should be done in order to standardize the LERV procedure.

## 1. Introduction

Common bile duct (CBD) stones are concomitant with gallstones in 10–15% of the patients, with the percentage of association between 8% and 15% in patients under the age of 60 and between 15% and 60% in patients over the age of 60 [1]. The standard treatment for concomitant CBD stones and gallstones remains debatable, and there is no consensus on the optimal management strategy. This could be due to multiple factors, including the level of suspicion for choledocholithiasis, preferences (patient and physician), resources, and the expertise of the surgeons, endoscopists, and radiologists [2].

The well-known and popular treatment options include (1) laparoscopic cholecystectomy after endoscopic treatment (a two-step procedure), (2) endoscopic treatment after laparoscopic cholecystectomy (a two-step procedure), (3) laparoscopic or open cholecystectomy and CBD exploration simultaneously, and (4) cholecystectomy and CBD exploration simultaneously using a transcystic approach [1–3].

The most commonly used minimal invasive technique in practice is the two-stage management, which consists of preoperative endoscopic retrograde cholangiopancreatography (ERCP), sphincterotomy, and CBD clearance followed by laparoscopic cholecystectomy. However, with this approach, many patients will be submitted to an unnecessary ERCP while others may develop complications, mainly pancreatitis, due to inadvertent pancreatic duct cannulation [4].

Several recent studies showed laparoscopic cholecystectomy with simultaneous intraoperative ERCP using the laparoendoscopic rendezvous (LERV) technique to be an optimal approach for concomitant gallstone and CBD stone as it offers a significant advantage over the traditional two-stage methods and by improving patient compliance and clinical results [5].

The LERV technique, which was first described by Deslandres et al. in 1993, is a combined procedure in which the gallbladder is laparoscopically removed while a CBD stone is simultaneously cleared endoscopically by selective CBD cannulation facilitated by laparoscopic placement of a guidewire, through the cystic duct, into the duodenum [6]. Cavina et al. [7] reported in 1998 that the LERV technique was an efficient method to treat choledocholithiasis and that there was no difference between the LERV technique and laparotomy or a combination of laparoscopic cholecystectomy and endoscopic sphincterotomy regarding the removal rates of CBD stones and the incidence of complications.

Recently published reports have indicated that evidence favoring the LERV technique is very promising and clearly demonstrates the main advantages of this technique in regard to the lower incidence of complications such as perforation, bleeding, and the significantly lower incidence of hyperamylasemia and post-ERCP pancreatitis. The selective cannulation of the CBD which avoids the inadvertent cannulation of the pancreatic duct is another advantage in addition to the contrast medium which is not injected retrogradely as during the traditional ERCP, when the medium may accidentally be injected under pressure into the pancreatic duct. The LERV technique is also associated with a reduced operation time, lower technical difficulties, shorter hospital stay, and less medical costs [8–16].

A literature review revealed many papers reporting the efficacy and advantages of the LERV technique in the management of concomitant cholecystocholedocholithiasis, but none had focused on its usefulness in tackling difficult cases, or patients presenting with difficult medical conditions. In this paper, we report our experience in performing the LERV technique to patients with unusual presentations in terms of anatomical difficulty, pregnancy, multiple comorbid diseases, and postlaparotomy and anesthesia phobia. We aim to highlight the effectiveness of the LERV technique in some clinical situations where conventional methods would fail or carry high risks in adequately managing concomitant cholecystocholedocholithiasis.

## 2. Methods

**Figure 1:**
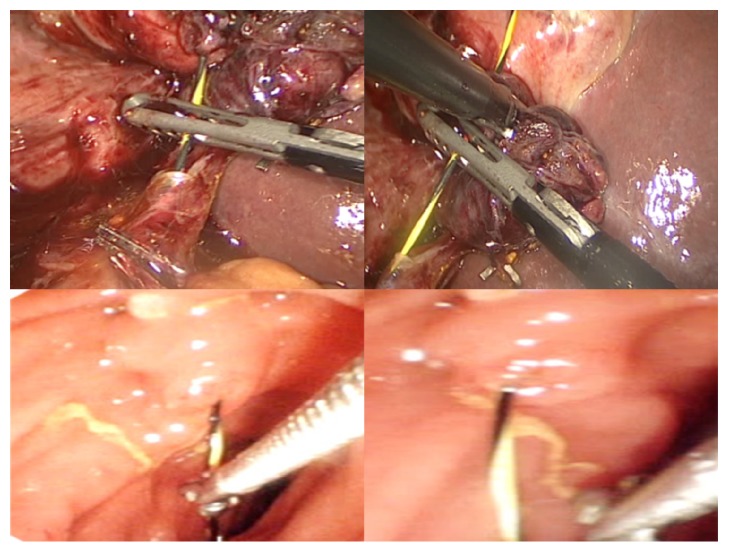
Intraoperative images showing laparoscopic guidewire insertion through the cystic duct by the surgeon and pulling the guidewire from the duodenum using biopsy forceps by the endoscopist.

**Figure 2:**
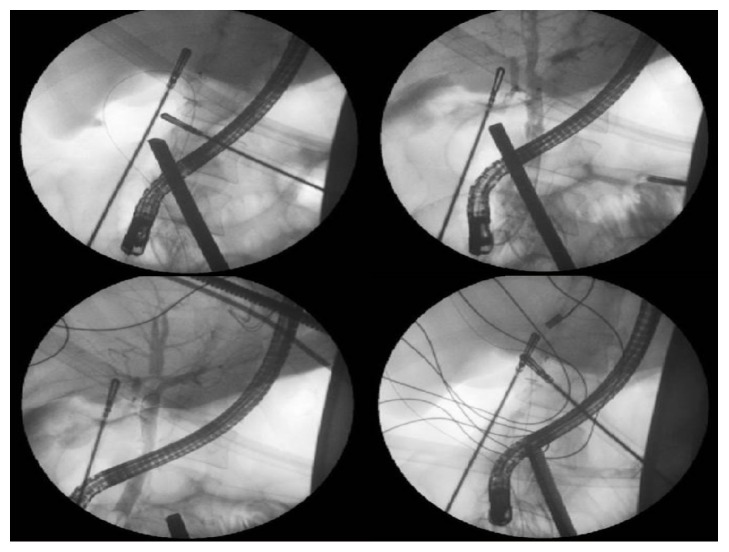
Fluoroscopy images showing guidewire rendezvous cannulation of the common bile duct.

**Figure 3:**
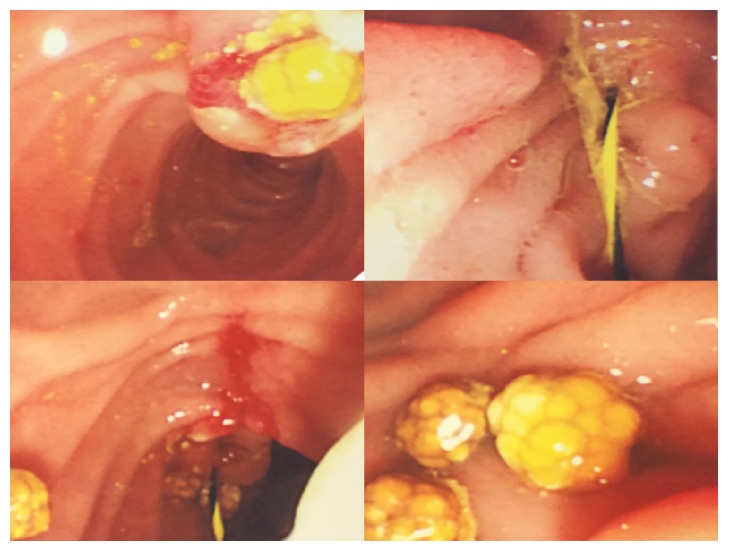
Intraoperative images showing sphincterotomy and insertion of the guidewire through the ampulla of Vater and stone removal by balloon sweep.

Our approach of the LERV procedure was carried out as follows: First, the patient was placed in the supine position. A standard laparoscopic cholecystectomy was started; dissection to identify the cystic artery and duct, ligation of the artery, and a small incision was made in the cystic duct. The guidewire was introduced from the medial subcostal port to allow horizontal insertion through the cystic duct. It was then advanced through the ampulla of Vater from the CBD into the duodenum, and the instruments were removed. Second, the endoscope was inserted by the endoscopist from the mouth to the duodenum, the guidewire was pulled out of the mouth, the sphincterotome was inserted, and an intraoperative sphincterotomy was done, followed by stone removal by a balloon, and the endoscope was removed. Third, the cystic duct was clamped and cut, the gallbladder was dissected from the liver bed and removed, and the laparoscopic cholecystectomy procedure concluded (Figures 1–3).

## 3. Patient 1: Anatomical Difficulty in Old Age

An 82-year-old female was admitted through the emergency room complaining of recurrent attacks of right upper quadrant pain of six-month duration. In the three days prior to presentation, she started to have increasing pain associated with vomiting. Examination revealed jaundice and a positive Murphy's sign. The patient underwent ERCP on the day of admission, but the procedure was aborted due to hypoxia and patient's desaturation. The following day, the patient was taken for a second attempt of ERCP under conscious sedation. There was difficulty cannulating the CBD due to anatomical difficulty and a knife cut was made above the ampulla but did not yield any bile. A week after admission, the patient was planned for a LERV procedure. During the laparoscopic cholecystectomy, the gallbladder was seen to be attached directly to the CBD. There was a small, short-necked cystic duct, and a catheter was introduced by the help of a grasper through the cystic duct down to the CBD down to the duodenum, and the guidewire was inserted through the cystic duct down to the ampulla. The procedure continued, and the balloon sweep resulted in the removal of three small stones. The procedure was completed after placement of the stent. The LERV procedure was well tolerated, and the patient was discharged home two days later without any postoperative complications.

## 4. Patient 2: Pregnancy

A 23-year-old female presented with a history of recurrent right upper quadrant abdominal pain and fever for two days. She had tenderness in the right upper quadrant and was found to be pregnant (6 weeks). The clinical situation was fully explained to the patient including the high risk of abortion. After being consented, she underwent ERCP, the ampulla was visualized, and it was very small and bulky with a possible stone in the submucosal area. The ampulla was cannulated, and contrast was injected showing dilated CBD, approximately 1.2 cm with distal CBD stricture. Several attempts to cannulate the CBD were unsuccessful; a needle knife cut was made. Two days later, the patient was taken to the operating room for a LERV procedure. Her abdomen was covered by lead. A low intra-abdominal pressure insufflation (12 mmHg) was maintained to decrease the risk of abortion. The procedure was completed successfully. Two days later, the patient was discharged home in a stable condition, with no effect on the pregnancy reported, and eventually, she delivered a full-term baby.

## 5. Patient 3: Multiple Comorbid Diseases

A 51-year-old female, known to have bronchial asthma, hypertension, congestive heart failure, and end-stage renal disease on regular hemodialysis 3 times per week, presented with a six-month history of progressively worsening recurrent attacks of right upper quadrant pain, with recent onset of fever and vomiting. Examination showed a positive Murphy's sign. She was started on intravenous antibiotics. Initial ERCP failed due to a hypotensive attack. Two days later, after controlling the medical conditions, she was taken to the operating room for a LERV procedure. The procedure was concluded by a placement of a stent. Four days later, the patient was discharged home in a stable condition.

## 6. Patient 4: Postlaparotomy and Sigmoid Resection

A 45-year-old patient, 1-year post sigmoid resection for diverticular disease, presented with right upper quadrant pain radiating to the back and right shoulder, nausea, vomiting, and passing dark urine. He had tenderness in the right upper quadrant. He also had a significant phobia of hospitals. The patient was admitted and started on intravenous antibiotics (Cefuroxime and Metronidazole). The plan was to perform the LERV procedure. Laparoscopic cholecystectomy was started by releasing and dividing extensive multiple abdominal adhesions, both liver lobes were pulled up to the anterior abdominal wall, and the gallbladder was grossly distended and very large in size and was retracted above the surface of the liver. The cystic artery and cystic duct were identified, and the artery was clipped and cut. A small incision through the cystic duct was made. A guidewire was passed through the cystic duct down to the CBD to the duodenum. The duodenoscope was introduced down to the stomach and then to the second part of the duodenum. The ampulla was visualized; the guidewire was pulled out (using biopsy forceps) through the scope. The injected contrast showed dilated CBD and multiple CBD stones. Sphincterotomy was done, and the guidewire was pulled out. A new guidewire was placed through the endoscope, and the balloon was inserted. Multiple CBD stones were removed, more than 8 stones, each approximately 0.5–0.8 cm. Duodenoscope was withdrawn, and the surgery was continued by removing the gallbladder from its liver bed using cautery and hook. Four days later, the patient was discharged home in a stable condition.

## 7. Results

All patients tolerated the LERV technique very well; no intraoperative occurrence was reported. Mean operative time was 86.3 ± 17.2 minutes; mean time of the endoscopic part was 29.4 ± 3.57 minutes. Mean blood loss was 44.3 ± 18.2 mL (range 20–85). Residual stone, postoperative complications, postoperative morbidity, and postoperative mortality were 0 (0%). Postoperative short hospital stay was reported in all patients, average 3 days (range 2–4). The results are summarized in Table 1.

## 8. Discussion

No incidence of postoperative pancreatitis was reported in our cases, which is in accordance with the clinical fact that LERV had reduced the risk of pancreatic ductal injection and cannulation and, hence, eliminated the risk of postoperative pancreatitis [6].

In 2013, Mauro et al. [11] published a study evaluating LERV in octogenarians and found it to be equal to younger patients in terms of cost-effectiveness, but it may lead to a higher conversion rate and longer hospital stay. In the first case, the hospital stay was 2 days, which is shorter than that of the multiple comorbid diseases and postlaparotomy and sigmoid resection patients and equal to the pregnant patient. No conversion was needed.

Some drawbacks and limitations were described in the literature about the LERV technique. The first drawback is the difficulty in some cases to manipulate the guidewire when passing it through the cystic duct and duodenal papilla. Tzovaras et al. [12] reported that, in 6 cases, conversion of LERV to traditional intraoperative ERCP was due to failure to advance the guidewire through the cystic duct into the duodenum due to the presence of anatomic variations. Secondly, in some patients advancing the guidewire through a spiral tortuous cystic duct is tedious and time-consuming; it is also quite difficult in some cases to control the direction of the guidewire and advance it through the duodenal papilla. Also, it may be time-consuming during the reciprocal maneuver of pushing and pulling the guidewire out of endoscope [13, 14]. Thirdly, intraoperative endoscopic sphincterotomy during the LERV technique is challenging as it requires two different teams, the surgical and the endoscopic teams, including the position of the patient on the operative table and the need for endoluminal insufflation for endoscopic vision. This implies a longer operative time (of about 60 min) than for laparoscopic cholecystectomy alone, and a longer hospital stay for the preoperative workflow [15]. Fourthly, bowel insufflation and bile leakage through the cystic duct during the procedure were considered drawbacks of intraoperative ERCP, which could be worse if the duration of ERCP is prolonged. Therefore, it is important to reduce the time of both the whole procedure and the endoscopic part [16]. The fifth drawback is the lack of endoscopic facilities in the operating room and the need for the endoscopic team to transfer and install their instruments, which is rather time consuming [16].

The incidence of gallstone disease and its related complications (biliary colic, acute/chronic cholecystitis, and biliary pancreatitis) in pregnant women was reported to vary from 1 to 8/10,000 per year [17]. Clinically, patients presenting in the first trimester should be treated initially with conservative therapy followed by laparoscopic cholecystectomy during the second trimester. For patients presenting in the second trimester, laparoscopic cholecystectomy can be offered in the same trimester. For patients requiring surgery in the third trimester, open cholecystectomy was considered a better option [18]. A literature review showed that laparoendoscopic single-site cholecystectomy in pregnancy was reported in three cases [19], but the LERV technique for concomitant cholecystocholedocholithiasis in pregnancy was not reported at all. To the best of our knowledge, our 23-year-old first trimester pregnant patient is the first ever reported case of successful LERV during pregnancy in general, and in the first trimester in particular. The patient was discharged safely in two days avoiding second admission and second sedation for laparoscopic cholecystectomy.

The development of intra-abdominal adhesions postoperatively between the abdominal scar and underlying viscera is a well-known consequence of laparotomy for major surgical procedures. It is clinically proven to consider patients undergoing laparoscopy after a previous laparotomy at an increased risk for the existence of adhesions between the old scar and the bowel and omentum [20]. In the fourth patient who had laparotomy and sigmoidectomy for diverticular disease, neither the midline scar nor the extensive intra-abdominal adhesions affected the procedure or outcome of the LERV technique. Careful, meticulous approach is the keystone.

A recently published article [21] suggested that in the case of patients with severe comorbidity rendering them unfit for surgery and those presenting with symptoms of CBD obstruction (jaundice, cholangitis, and recurrent acute pancreatitis), performing a single step ERCP without cholecystectomy (leaving the gallbladder in situ) may be the safest option, but in that study, patients with cholecystitis were not included. Our experience in managing the third patient showed that the multiplicity of the advanced comorbid diseases did not preclude or adversely affect a successful LERV. This observation leads us to agree with the statement that “the choice of the best strategy is often led by the local presence of professional expertise and resources, rather than by a real superiority of one strategy over another” [22].

## 9. Conclusions

We conclude that the LERV procedure is a safe and effective treatment option for the management of concomitant cholecystocholedocholithiasis, even in difficult situations where other methods would fail or carry high risks, or in patients presenting with difficult comorbid diseases and clinical conditions including pregnancy. This procedure may emerge as an attractive alternative option for high-risk patients. The LERV procedure is associated with significantly lower postoperative complications, shorter hospital stay, and lower medical costs. A patient's wishes may also influence the selection of this procedure. More scientific studies recruiting more patients with other difficult presentations should be done in order to standardize the LERV procedure.

## Figures and Tables

**Table 1:** Personal, clinical, laboratory, and radiological data of the study patients.

Patient	Age	Gender	Difficulty	Bilirubin	AST	ALT	Amylase	Alkaline phosphatase	Gallbladder stone	CBD stone	CBD diameter
1	82 Y	Female	Anatomical difficulty in old age	75 *μ*mol/L	266 IU/L	181 IU/L	147 IU/L	354 mmol/L	Multiple small	Single 0.8 cm	1.3 cm
2	23 Y	Female	First trimester pregnancy	90 *μ*mol/L	186 IU/L	490 IU/L	69 IU/L	200 mmol/L	Multiple small	Multiple small	1.2 cm
3	51 Y	Female	Multiple comorbid diseases	35.7 *μ*mol/L	183 IU/L	297 IU/L	78 IU/L	210 mmol/L	Single 1.1 cm	Single small	1.8 cm
4	45 Y	Male	Postlaparotomy and sigmoid resection	33.4 *μ*mol/L	639 IU/L	144 IU/L	34 IU/L	187 mmol/L	Multiple small	Multiple small	1.4 cm

## Abbreviations

LERV: Laparoendoscopic rendezvous
ERCP: Endoscopic retrograde cholangiopancreatography
CBD: Common bile duct
AST: Aspartate aminotransferase
ALT: Alanine aminotransferase.
